# EXAMINING SEX DIFFERENCES WITHIN THE RELATIONSHIP BETWEEN PHYSICAL ACTIVITY AND EXECUTIVE FUNCTION IN OLDER ADULTS

**DOI:** 10.1093/geroni/igz038.1915

**Published:** 2019-11-08

**Authors:** Genna Losinski, Hilary J Hicks, Alex Laffer, Amber Watts

**Affiliations:** University of Kansas, Lawrence, Kansas, United States

## Abstract

Research has demonstrated sex-associated differences in physical activity and its benefits on cognition in older adults. The present study explored differential associations between moderate-to-vigorous physical activity (MVPA) and executive function, which is known to decline with aging. N = 53 older adults without cognitive impairment (M = 73.19 years, SD = 6.53) wore accelerometers (Actigraph GT3X+) during 7 consecutive days. Activity intensity was categorized as light, moderate, or vigorous based on Freedson Adult Vector Magnitude cutpoints. Participants completed a battery of executive function tests: Digit Symbol Substitution Test, Verbal Fluency, Trail Making Test, and Stroop Color-Word Test. A cognitive composite score was created using confirmatory factor analysis. Women had a higher mean MVPA (4.57%) than men (2.64%, t (19.04) = -2.49, p = .022). However, executive function performance did not differ by sex (t (26.20) = 1.67, p =.107). The interaction between sex and time in MVPA did not predict performance on executive function, adjusting for age and education. Older age was the only significant predictor of poorer executive function (β = -0.038, p = .003). The current sample had limited engagement in MVPA (range 0.18-10.87%). These findings suggest that the amount of engagement in MVPA in a free-living environment may not be sufficient to demonstrate sex-associated differences in executive function performance. Future studies should explore executive function performance with other intensity levels and examine other areas of cognition.

